# Finance is good for the poor but it depends where you live

**DOI:** 10.1016/j.jbankfin.2012.04.022

**Published:** 2013-05

**Authors:** Johan Rewilak

**Affiliations:** Department of Economics, University of Leicester, Leicester LE1 7RH, United Kingdom

**Keywords:** Financial development, Economic growth, Poverty

## Abstract

I examine whether or not the incomes of the poor systematically grow with average incomes, and whether financial development enhances the incomes of the poorest quintile. Following the methodology of Dollar and Kraay (2002), I find, once extending Dollar and Kraay’s data, their findings are robust to the Lucas critique and economic growth is important for poverty reduction universally. However, in comparison to other authors’ work I show financial development aids the incomes of the poor in certain regions, whilst it may be detrimental in others. This proposes evidence against a “one size fits all” model adding a further contribution to the literature on financial development and poverty.

## Highlights

► Examine the impact of economic growth and financial development on poverty. ► Extend the Dollar and Kraay (2002) study and test the results for the Lucas critique. ► Economic growth is important for poverty alleviation universally. ► Financial development exhibits regional differentiation for poverty reduction.

## 1 Introduction

Since the turn of the millennium, and up until the financial crisis, growth of the world economy has been relatively strong. Growth with equity is a challenge that most governments have tried to establish with sceptics suggesting both cannot be accomplished simultaneously. Dollar and Kraay (2002) in an influential paper asked; “does the per capita income growth of the poor rise proportionally, less than proportionally, or more than proportionally to average per capita income growth?” their findings suggest that this is the case, hence emphasise the importance of economic growth for poverty reduction.

If growth is good for the poor, then growth enhancing policies should be encouraged. Literature suggests that certain policies and institutions exist that may further stimulate economic growth. In their paper, Dollar and Kraay suggest that trade openness, government consumption, the inflation rate, the rule of law and financial development may influence economic growth. Furthermore, a claim laid down is that these policies may even accrue or offset the income growth of the poor.

This is not to suggest that further factors may influence the economic growth process. Education is one tool that has been attributed to growth amongst others.

A rough battery of empirical evidence supports Dollar and Kraay’s suggestions, in which openness to trade has been found to increase long run GDP per capita growth. Using the Sachs Warner index as a measure of openness, Greenaway et al. (1998) find that when this indicator variable takes the value of one highlighting an open economy, growth may be increased by 46%. Easterly and Rebelo (1993) report that government consumption is harmful to growth; however, Dowrick (1996) shows that government consumption may be growth enhancing if it is maintained between a region of 10–18%. There is substantial evidence that inflation is harmful to growth. Barro (1996a,b) finds that an increase in inflation of ten percentage points retards growth by 0.2–0.3% hence over a thirty year period growth may be reduced up to 7%. Examining past work on the role of strong property rights and/or rule of law Knack and Keefer (1995) mention their importance for growth while Barro (1996a,b) empirically tests this hypothesis finding a strong legal system is required for favourable growth rates.

The literature on financial development and economic growth is extremely rich where early theoretical suggestions such as those by Schumpeter (1911) highlight the importance of finance for economic growth. Critics have challenged this view, suggesting finance merely follows growth, Robinson (1962). King and Levine (1993) in their interestingly titled paper “Schumpeter might be right” test these theories empirically and find that finance may cause economic growth. Moreover, the paper’s results have since been complemented by further studies, including time series approaches and those of panel data from authors such as Arestis and Demetriades (1997), Luintel and Khan (1999), Levine et al. (2000) and Levine (2003).

Recently, Rousseau and Wachtel (2005) examine whether or not the finance-growth nexus has become extinct. The authors take the King and Levine (1993) data and thoroughly examine the robustness of this relationship finding that the results fail to carry over when more data is added to the research question. On closer inspection they find, when splitting the sample into 5 year periods, the 1970s and early 1980s were the main drivers of the relationship, hence from 1990 onwards the data was susceptible to the Lucas critique.

If financial development is no longer growth enhancing as the results from Rousseau and Watchel seem to suggest, a question emerges; does financial development still benefit the poor?

If finance is available to the poor, then it may provide the poor with a means to save. In less developed countries (LDCs) cases exist where money is stored under a mattress, which may be problematic and hamper a households ability to move up the social ladder. First, this money is vulnerable to theft, and keeping track of where all the money is hidden within a household is challenging. Second, during periods of macroeconomic instability, which may include periods of hyperinflation, savings accounts which are indexed to inflation may prevent this money from eroding away in value, a benefit for the poor. With a lack of savings accounts the poor may waste accumulated assets on the purchase of unnecessary physical capital, for example oxen for farming. These physical assets do not improve productivity or offer any major returns to the poor; they are just purchased for their ease of monitoring/storage and are highly illiquid when acquired. Moreover the presence of savings accounts may prevent transitory poverty by providing opportunities to utilise savings and consumption smooth during difficult times.

Furthermore, savings accounts in financial institutions may help the poor as accumulated savings over a generation may allow a family’s offspring to pay for, and attain higher levels of formal education if parents are altruistic. This allows inter-generational mobility through the classes to be established more easily.

If we assume a fixed cost to be an entrepreneur, with perfect financial markets, a poor entrepreneur could go to a bank, highlight his business plan, and the ability of financial institutions to monitor and recognise good investments may allow poor entrepreneurs (those with the greatest entrepreneurial ability and the most talent) to have society’s funds directed to them, as opposed to those with average ideas and existing wealth/established connections/collateral to take out a loan. This provides the necessary opportunities for the poor to move up the social ladder.

Research on finance and poverty alleviation is more recent and in its infancy compared to studies on finance and aggregate growth. Claessens and Perotti (2007) provide a summary of the existing literature, where Beck et al. (2007) discover fascinating empirical results.

Beck et al. (2007) complement the study of Dollar and Kraay (2002) with a stricter focus on the impact of financial development on poverty, specifically examining the Gini coefficient, the income share of the poor, and the percentage of people living on less than $1 a day.^1^ Their conclusions indicate that financial development is poverty reducing. Furthermore, they find that 40% of income growth from the poorest quintile is a result of reductions in inequality, but 60% due to the impact of financial development on aggregate growth. Hence, not only is financial development in their study positively associated with income growth of the poor, but their results suggest, that financial sector reforms, which reduce market frictions may also lower inequality, without the incentive problems which redistribution schemes that include generous social security payments create.

Hence I do not just focus on finance and its effects on poverty, but I consider whether or not aggregate growth has an impact on the poor in tandem. The motivation of this study is to examine first whether, unlike the results found by Rousseau and Watchel on the finance-growth nexus, do Dollar and Kraay’s (2002) findings remain with the inclusion of more data.

Second, I complement the Beck et al. (2007) study by using additional measures of financial development such as those used by King and Levine (1993) which were found to break down by Rousseau and Wachtel (2005) when modern data was included. Moreover, I choose to include a market based measure of financial development in the hope to prove that for poverty reduction it is just the overall level of financial development that matters, regardless of whether the development comes from the bank side or the market side. In addition, I choose to strictly follow the Dollar and Kraay methodology in the hope that the relationship between finance and poverty proposed by Beck et al. (2007) withstands further scrutiny.

This study, when including further data covers over one hundred countries and spans over fifty years. I expect to find that growth is good for the poor, and my results are at least as significant as those provided by Dollar and Kraay (2002). Furthermore, I aim to add to the Beck et al. (2007) study and show that financial development is imperative to the income growth of the poor, irrelevant of the financial development indicator used.^2^

## 2 Data and methodology

The original data is from Dollar and Kraay (2002), available to download from The World Bank.^3^ The extended dataset comes from World Bank databases with information and definitions found in Table A of the appendix.

The dependent variable income growth of the poor is measured as the GDP per capita growth of the income of the lowest quintile.^4^ This measure is used as it is consistent with the study of Dollar and Kraay, which I am trying to extend and check who’s initial results hold, but also because it is a variable that is abundant.^5^

Financial development in this instance is measured as the depth of the financial system. Ideally, further measures that show the outreach of the financial system (breadth) would be useful, for example data showing the amount of access the finance system provides, but sadly due to data scarcity this cannot be accomplished. Private Credit as a ratio of GDP is one of the most frequently used measures of financial development and measures the channelling of savers’ funds to private projects, one main function of financial intermediaries. This variable was used by Beck et al. (2007) in their own particular extension of Dollar and Kraay.

Further measures of financial development are also well used in the literature. King and Levine (1993) use Liquid Liabilities as a ratio of GDP.^6^ This variable was found to be significant in the study of King and Levine (1993) on aggregate growth but became insignificant in the Rousseau and Wachtel (2005) paper when they extended the former authors’ data. Hence I choose to include this measure of financial development due to the interesting experiences this variable has shown in the literature.

I incorporate a market measure of financial development. The chosen variable is Stock Market Capitalisation. Empirical results suggest that stock markets may increase growth, Levine and Zervos (1998), with further conclusions from the authors highlighting that banks provide different services than those provided by stock markets stressing their importance. Moreover, research suggests that countries with better developed stock markets also have better developed banks. In entrepreneurial projects where disagreement exists about investing in a venture, a well financed minority of investors may still be able to finance the project through the purchase of shares, where a bank may be reluctant to invest without a clear majority in agreement of funding the enterprise. This may be important for poor entrepreneurs, who may only be able to convince a minority of investors of their project. Other research states that the overall development of the financial sector is important regardless if the development is from banks or markets, furthermore highlighting the inclusion of this variable.

Ideally, a measure for the stock market such as the Turnover Ratio, or even Value Traded, would have been appropriate to use in the study as this shows the liquidity of the stock market. One important factor of trading in the stock market is that for a saver, a saver’s stocks may be transferred into cash quickly; however, as data is limited, the measure Stock Market Capitalisation as a ratio of GDP is used.^7^

The control variables selected in the study are those used by Dollar and Kraay (2002) and are selected here to make sure that, when their results are checked for the Lucas critique, everything remains consistent. Beck et al. (2007) favoured a different approach where they replaced the rule of law variable with the average years of school attainment to control for human capital and as opposed to controlling for GDP per capita growth at the mean level as Dollar and Kraay (2002) and I do, they control for GDP per capita growth using growth of the lowest income share.^8^

I measure the income of the poor as the income share of the lowest quintile. When updating the sample, if data from the same named source exists and is updated for country (*i*), I use that source irrelevant of quality ratings. If the data from the same named source does not exist for future waves, I select the observation based on two certain criteria: I try and choose the observations with the highest quality ratings while simultaneously trying to select the data sources that are most frequently used in the already existing dataset from Dollar and Kraay (2002).

The new data for the remaining variables was selected following the Dollar and Kraay (2002) procedure, where I select the last observation for a particular cross section (*i*) used by Dollar and Kraay, and then move forward a minimum of 5 years, selecting data for the next decade until time expires for that particular cross section. In some cases a particular cross section permitted the inclusion of more than one additional time period (*t*), as revisions in data meant that some data in the penultimate decade were now available. Hence, at times, two or three waves were added to certain cross sections, but this was a rarity.^9^

Table 1 shows how the number of observations and cross sections increase when I add further data while examining the financial variables.^10^ The far larger number of observations and cross sections in my study provides greater worldwide representation in my estimations and increased flexibility for the number of instruments used when using a System Generalised Method of Moments (GMM) estimator. Furthermore, the Dollar and Kraay data spans across four decades with observations from 1956 to 1999. The additional wave(s) include observations until 2008, thus further modernising this study.

An important observation is that when Beck et al. (2007) run their system estimator using Private Credit, they have 245 observations whilst I have close to 300 when a full set of controls are imposed, hence I have a richer dataset. The data also spans into a time when the world economy suffered a shock with the global financial crisis. Despite not being my primary research question, it may be interesting to see whether or not the crisis did have any effects on the income growth of the poor in comparison to Beck et al. (2007).

Initially, I carry out cross country regressions where I regress the per capita income of the poor on the natural logarithm of average per capita income. Unlike other methods, which use one observation per cross section, I use all available data in order to preserve degrees of freedom, hence run pooled cross country regressions.^11^

Eq. (1) is initially estimated using ordinary least squares and then using instrumental variables. Instrumenting for mean income is carried out by using growth in mean income prior to time (*t*).

(1) $$Y_{i\text{,}t}^{p}=\alpha +\beta Y_{i\text{,}t}+\mu_{i}+\epsilon_{i\text{,}t}$$

(2) $$Y_{i\text{,}t}^{p}-Y_{i\text{,}t-k}^{p}=\beta (Y_{i\text{,}t}-Y_{i\text{,}t-k})+(\epsilon_{i\text{,}t}-\epsilon_{i\text{,}t-k})$$

It would be quite common to find unobserved country specific effects (*μ*_*i*_) to exist such as those in Eq. (1), hence I regress Eq. (1) in differences, Eq. (2). I expect individual country effects to have some influence on the results, where certain countries located in advantaged regions and abundant with resources may have a positive effect on the income growth of the poor, but to what extent I cannot be sure. Nevertheless differencing sweeps away these individual effects.^12^ I then estimate Eq. (2) using ordinary least squares and then an instrumental variable approach. When applying the instrumental variable estimator to Eq. (2), a further instrument is used, the level of mean income at the beginning of the period.

Despite removing the unobserved country specific effects by estimating the equation in differences (2), it would be more ideal to exploit the wider cross country variation as opposed to the time series variation as Eq. (2) does. As a result I use a panel estimator and the favoured estimator is System GMM proposed by Blundell and Bond (1998). If we assume that the coefficients of (1) and (2) are the same, we may regress our relation as a system. The proposed estimator not only manages to fully control for country-specific effects, but also may deal with endogeneity concerns.

(3) $$Y_{i\text{,}t}^{p}=\alpha +\beta Y_{i\text{,}t}+\gamma^{\prime }X_{i\text{,}t}+\mu_{i}+\epsilon_{i\text{,}t}$$

(4) $$Y_{i\text{,}t}^{p}-Y_{i\text{,}t-k}^{p}=\beta (Y_{i\text{,}t}-Y_{i\text{,}t-k})+\gamma^{\prime }(X_{i\text{,}t}-X_{i\text{,}t-k})+(\epsilon_{i\text{,}t}-\epsilon_{i\text{,}t-k})$$

Eqs. (3) and (4) introduce additional variables into the specification in the (*X*) matrix. Some of the control variables may be endogenous. Dollar and Kraay claim endogeneity concerns may exist for financial development and inflation, but not openness to trade.^13^^,^^14^ As I strictly follow Dollar and Kraay (2002), I instrument for income only, as my results, as those found in Dollar and Kraay (2002), show the tests of overidentifying restrictions pass even when instrumenting for income only. This provides indirect evidence that the *X*variables are uncorrelated with the error terms. Second, if I was to instrument for all possible endogenous variables using appropriate lags, then GMM may become inconsistent.^15^

The GMM estimator controls for endogeneity using internal instruments where it uses specified lagged variables in level terms as instruments for the regression in differences, and in the level equation, chosen lagged differences are used as instruments.

The chosen estimator requires that there exist more instruments than endogenous regressors, hence the equation is over identified.^16^ Two specification tests exist to check the validity of the instruments, the Hansen *J*-test or the Sargan test. A second assumption is required when using this estimator that no second order serial correlation exists; however, the estimation procedure requires the presence of first order serial correlation. If these two main assumptions are not violated, hence both null hypotheses are not rejected from the specification tests, then the coefficient estimates are efficient.

In all the regressions, I run a hypothesis test to see if growth is good for the poor. This follows suit to Dollar and Kraay, where I test whether the coefficient on average per capita income is 1. If the coefficient on (*β*) is not significantly different from 1 then the incomes of the poorest quintile grow systematically with average incomes- a result that I wish to hold for all the estimations.

## 3 Results

Table 2 shows the replicated results of Dollar and Kraay using Stata where Table 3 shows the results when the data is updated. In all specifications it is seen that average growth is positive and significant with a coefficient close to 1. Table 2 suggests that the null hypothesis of income growth of the poor being proportional to mean income growth, is only rejected in column one. However this rejection seems to be a positive rejection where a 1% increase in mean income growth would increase growth of the poorest quintile by more than 1%.

When the data is extended, the results appear to be stronger. For the benchmark case, it may be stated that growth is good for the poor, and that the original results from Dollar and Kraay (2002) are robust to the Lucas critique. With the additional data, the specification test that (*β* = 1) is rejected on three occasions, in columns one, two and five of Table 3. Moreover, in all three instances, the nature of the rejection posits that income growth of the lowest quintile grows more than proportionally to average per capita income growth.

One variant of the specification is to test whether the slope of average growth on the system estimator varies by region. The Dollar & Kraay results show that for most regions, the overall effect of the coefficients is approximately one; however, the coefficient for the omitted category, the developed countries, shows an elasticity of 1.35%.

The elasticity of the poors’ incomes with respect to average incomes in Latin America is 0.34, which is very low, and Dollar and Kraay (2002) state this result is attributed to the unusually poor performance of instruments in the sample.

When the sample is extended, the results differ by a large degree. Here with the addition of a decade’s worth of data, the growth coefficient for the control group shrinks to over half its size, and the slope coefficients for the regions switch signs.^17^ Moreover, the overall effects exhibit interesting results, as in the new sample all the coefficients with the exception of Sub-Saharan Africa are very close to unity, and are all significant. If we compare that to the control group, it is shown, that if growth rates for all countries were equal, a catching up effect in terms of income growth would take place for the poor in those regions relative to the control groups poor.

It may be plausible to suggest that the results seen in Table 4 have occurred due to the intensity of mean economic growth as a determinant on the income growth of the lowest quintile. Over the period from 2000–2009, when the sample has been updated, with the exception of the financial crisis, most regions have had growth rates that have outperformed those of the control group. It may be for that very reason that the results exhibit a catching up effect for the income growth of the poor relative to the control group.

This highlights an important consideration, as it may be the case that when examining financial development, the effects may differ between the control group, sub-Saharan Africa, and all remaining regions just as they do with the growth regressions in Table 4.

Beck et al. (2007) tested the relationship between financial development and the level of economic development, finding that this interaction was insignificant, hence income growth of the poor did not vary with the level of GDP per capita. I on the other hand, posit that regions may act differently to financial development and apply the idea that locality and space may be more influential than varying levels of GDP per capita alone.

The inclusion of control variables to vary the specification yield results similar to Dollar and Kraay (2002). Included are trade openness, government consumption, the presence of inflation, and the rule of law quality. These variables are included one at a time following the authors’ methodology, and the results in Table 5 show all the coefficients are correctly signed, with similar magnitudes as in the Dollar and Kraay paper.

In all regressions, average GDP per capita remains positive significant and yields a coefficient close to one indicating that the relationship between mean income growth and that of the income growth of the poor is robust. Moreover, both the tests for overidentifying restrictions and no presence of second serial correlation are passed with their respective *p*-values in the non rejection zone of the null hypotheses.

I show the effects of finance on the poor in Table 6. Here columns 1–4 present the results for the original sample size and columns 5–8 for the extended period. The variable Commercial Bank Assets as a ratio of Total Bank Assets is successfully replicated in column one using the Dollar and Kraay data. This variable is positive and insignificant, which is also the case when the sample is extended. The addition of Private Credit in column 2 yields a coefficient which is also positive and insignificant, one main difference to the results found by Beck et al. (2007). In their study, the authors found Private Credit to be a significant determinant to the income growth of the poor, and the same applied for their other measures of poverty. In the extended sample which covers the Beck et al. (2007) time period Private Credit enters insignificantly. This seems to suggest that the relationship between financial development and poverty alleviation is sensitive to the specification chosen to examine poverty.

When examining further measures of financial development, Liquid Liabilities enters positively and significantly. This reflects the overall size of the banking sector in relation to economic activity. Here in both sample periods the results are significant and the coefficient is greater when the larger data set is used; however, both coefficients are in line with previous estimates of the literature with coefficients of approximately 2%. The variable Stock Market Capitalisation is negative and significant in column 4, indicating finance is detrimental to income growth of the poor, but becomes insignificant when the sample is lengthened.^18^

The results indicate that past findings from the finance and poverty literature are susceptible to the specifications and control variables used, but also to the measure of financial development. Here, Private Credit, which shows the financial resources provided as credit to the private sector, is insignificant. Yet, when Liquid Liabilities is used as a financial development indicator, the results are significant. It may be that what the poor really require are deposit accounts, or methods to save money which Liquid Liabilities may pick up more effectively than Private Credit. Hence, the results suggest that opportunities to save may matter more for the poor, as opposed to borrowing opportunities to expand businesses or become entrepreneurs.

Once more all the specification tests are passed, and examining the coefficient on mean growth, it keeps its strong significance and the hypothesis of (*β* = 1) is never rejected.

Table 7 takes the results from Table 6 further interacting the financial development variable with worldwide regions. Beck et al. (2007) mention how over their sample period the population living on less than $1 a day in Thailand fell dramatically, but how the rate doubled in Venezuela, and how certain countries located in certain regions experienced large increases in their Gini coefficients whilst others noticed a fall. Moreover, I choose to examine regional variation as I am motivated by ideas of spatial economics, where contiguity of countries that share borders may be categorised more closely than by mere economic development. The results in Table 7 show that, depending on which region of the world a country is situated, it has a severe impact whether financial development is good for the income growth of the poor. More importantly, three of the financial development indicators all now enter positively and significantly providing evidence that a “one size fits all” model may not be the case when examining finance and the poor, an additional contribution to the existing literature.^19^

Foremost all the banking sector measures of financial development enter positively and significantly for the control group.^20^ The market based measure Stock Market Capitalisation is the only financial variable that is insignificant.

Examining the interactions between financial development and the regions, the first bank based measure Commercial Bank Assets has significant interactions for the Latin America & Caribbean region and the Sub-Saharan African region. When testing for the linear combinations this financial measure then returns a positive and significant coefficient for Eastern Europe & Central Asia, and for South Asia.

Examining additional measures of finance, Private Credit returns a regression with all the interactions significant. When testing for their linear combinations, Eastern Europe & Central Asia, and South Asia suggest that an increase in Private Credit in these regions, may increase growth of income of the poorest quintile. The Latin American region and Sub-Saharan African region suggest that finance may be negatively associated with incomes of the poorest quintiles, where an increase in Private Credit will lead to reductions in income growth of the poor in those areas.

Liquid Liabilities has significant regional interactions for all areas except for the Middle East & North African zone. When testing for the linear combinations, all the regions maintain their significance with one exception, the area of Sub-Saharan Africa.

Column 4 containing the financial development variable Stock Market Capitalisation shows that finance is insignificant in the control region, Eastern Europe & Central Asia, and in the Middle East & North Africa. When the linear combinations are tested, a further region, East Asia & the Pacific, loses its significance. The remaining significant regions, Sub-Saharan Africa and Latin America and the Caribbean suggest finance is detrimental on income growth of the poor. The only remaining positive and significant region is South Asia where an increase in Stock Market Capitalisation may result in an increase in income growth of the poor.

The results show wide variation in the effects of financial development on the income growth of the poor between regions. The interesting question is why is this the case? It may be seen that, in general, finance has been extremely good for the income growth of the poor in South Asia and the Control group, while this is the contrary in Latin America. There is weak evidence from the results suggesting that finance is also harmful in Sub-Saharan Africa, whilst beneficial in Eastern Europe & Central Asia.

There are several possible hypotheses that may explain why finance has been fruitful in some regions and not others.

First, it may be that despite a deep financial sector, access is not universal. In India between 1977–1990, the Indian Social Banking Experiment took place where rural poverty fell dramatically, Burgess and Pande (2005). Here policy stated that a commercial bank could only open a branch in a location with existing bank branches, if it opened branches in four locations with no bank branches. The benefits to the poor were great; hence, in terms of policy, it may be that governments should focus on providing opportunities to expand financial access as opposed to purely focusing on depth. This should result in only those who voluntarily exclude themselves to be absent from the financial sector.

In the control region political pressures normally result in wide access and allow the poor to access finance; however, in regions such as South Asia where finance has been fruitful, past government policy broadening access may be the reason why the poor benefit from finance unlike in other regions such as Latin America & the Caribbean.

Second financial illiteracy of the population coupled with exploitive predatory behaviour of lenders may drive the results from Table 7. The poor may only require simple transaction accounts to take part in a market economy, but, being financially illiterate they may be provided with or ask for checking accounts, where severe overdraft charges may be incurred when the timing of payments goes wrong. It may also be a case where the naive poor are taken advantage of by predatory lenders who do not inform the borrower of all the conditions of the loan, crippling the poor with spiralling charges, where the poor may have been better off not participating in the financial sector. Educational advice such as teaching sound money management and legal systems that strictly enforce caveat emptor may prevent these adverse effects from occurring, as would stringent regulatory policy designed to make sure financial intermediaries do not abuse their position of power regarding their services.

Financial liberalisation is associated with increased competition, privatisation and foreign ownership, which may expand financial access for the poor once it becomes unprofitable to lend to existing wealthy clientele, Gormley (2004) and Mian (2006). In certain worldwide locations it may have taken longer for profit to dry up when serving existing clients, hence a disparity exists for why finance is beneficial in some regions as opposed to others.^21^ However, Ang (2010) found liberalisation in India specifically led to a worsening of the income inequality problem, where as the region of South Asia in this study, which composes of India, showed great gains from financial development. Hence there may be an argument that forced liberalisations or hasty liberalisation may be detrimental to the income growth of the poor, many of which were common in Latin America during the sample period. The results presented here on financial development indicate a “one size fits all” model may not be accurate of the world. These results may carry over for financial liberalisation, hence policy advice would be to plan country specific liberalisations and not base any plans on experiences of countries that have previously liberalised their financial systems. Moreover, the issue of financial instability may be playing a role where Akhter and Daly (2009) found in their study that if financial instability accompanies financial development, then this instability is detrimental to the well being of the poor. It may be that some regions faced this financial instability when developing their financial systems as opposed to others which may be an additional factor driving these results. These hypotheses, however, should perhaps be researched more in their own right.

Differences in the efficiency of the financial institutions may partially explain the results from Table 7. In regions where finance has benefited the poor it may be that loans were targeted at good enterprises that have grown, increased formal sector jobs (which in turn can provide poor individuals documentation to open their own financial accounts – a barrier in many LDCs), increased wages, hence benefited the poor. This may be as opposed to in Latin America & the Caribbean where lending has occurred to badly targeted SMEs who have not grown as expected, hence limiting the opportunities of the poor and not providing any income growth prospects for the lowest quintile. This leads us to financial policy advice suggesting that banks should perhaps implement greater screening of clients’ business proposals prior to lending.^22^

Finally, country specific effects of big-players within regions may influence the results. If theories of spatial economics are correct, it may be the case that neighbouring nations may adopt similar practices to the big-players, and as a result, individual specific effects from a country may have transmitted into the regions. This certainly may explain why Latin America may have a negative coefficient attributed to it, whilst South Asia has a positive coefficient. In Latin America, Brazil is the largest national economy, and where financial development has increased over time, poverty and inequality have remained relatively high despite their notable decreases. On the other hand India, the so called big-player in South Asia, has experienced a huge decline in inequality and poverty with a fairly stable growth in financial development. These questions I leave unexamined for future research.

## 4 Conclusion

Adding new waves of data to the existing research undertaken by Dollar and Kraay, I complement their findings that average incomes of the poorest quintile in a country rise and fall proportionally with average incomes. The addition of pro-growth policies in my estimations are also robust to the scrutiny of new additional data. It may be stated that governments that seek low levels of inflation, pursue open trade regimes, strengthen their legal systems and curb their government spending will create good platforms for average income growth. As income growth for the poorest fifth in society grows proportionally with average income growth, the results suggest to alleviate poverty by raising the per capita incomes of the poorest quintile; basic growth-enhancing policies still have a role to play.

Specifically focusing on finance and using further measures of financial development, I find financial development may alleviate poverty, but not universally. It is imperative to realise a “one size fits all” model does not work as different regions react differently to financial development when we consider income growth of the poorest quintile. The extreme variation can be specifically seen comparing South Asia, where financial development is successful in raising the income of the poorest quintile, with the region of Latin America & the Caribbean, where evidence suggests the contrary.

Governments may be required to intervene to promote financial provision for the poor, for at least the short term. Examples of schemes to increase outreach in the short run may be to use the already existent postal network to provide finance by extending the post office’s services. Here, high fixed and sunk costs have already been spent, so financial transactions may be provided at marginal cost. This should overcome the difficulties of providing for low income clients who only require small transactions until the private sector is ready to cater for them. This may occur once technological innovation has advanced to make it profitable to do so, or when already existent revenue streams have dried up serving high net worth customers.

I highlight that the poor profit as much as everyone else to overall per capita growth universally, but, with respect to financial development, this is not the case and certain regions respond better than others.

## Figures and Tables

**Table A:** Variable information.

Variable name	Definition	Source
Income of the poor	Measured as the share of income of the lowest quintile. Calculated by taking the share of income of the poorest fifth and then multiplying by GDP, and then taking the logarithm of this value	Dollar and Kraay (2002) which consists of a combination of sources, Deininger and Squire (1996), UN-WIDER (2000), Ravaillion and Chen (2000) and Lundberg and Squire (2000), IEP (2010). Additional waves are from UN-WIDER (2010)
GDP	This is the natural logarithm of real GDP per capita. It also appears in the body of the text as “average per capita income”	Dollar and Kraay (2002) sample and the World Bank, World Development Indicators (2010)
Openness	Exports and Imports added together as a ratio of GDP	Dollar and Kraay (2002) and The World Bank, World Development Indicators (2010)
Government share	Government Consumption as a ratio of GDP	Dollar and Kraay (2002) and The World Bank, World Development Indicators (2010)
Inflation	Calculated as one plus the inflation rate, and then the natural logarithm of this number is taken	Dollar and Kraay (2002) and The World Bank, World Development Indicators (2010)
Rule of law	An index composed to assign values for the quality of rule of law. Higher numbers indicate a stronger rule of law	Dollar and Kraay (2002) and the The World Bank WGI database (2010)
Commercial bank assets	Calculated as Commercial Bank Assets as a ratio of Total Bank Assets	Dollar and Kraay (2002) and the World Bank, World Development Indicators (2010) and the Financial Structure Database (2010)
Private credit	Calculated as Private Credit as a ratio of GDP, and measures the depth of the financial system, for example the financial resources provided to the private sector	The World Bank, World Development Indicators (2010) and the Financial Structure Database (2010)
Liquid liabilities	This is calculated as a ratio of GDP and measureasn the size of the financial intermediaries	The World Bank, World Development Indicators (2010) and the Financial Structure Database (2010)
Stock market	Calculated as the Stock Market Capitalisation as a ratio of GDP	The World Bank, World Development Indicators (2010) and the Financial Structure Database (2010)

**Table B:** Descriptive statistics of the main variables.

Variable name	Obs	Mean	Standard deviation	Minimum	Maximum
Income of the poor	568	6.830	1.283	3.369	10.07
GDP	568	8.033	1.164	4.740	10.76
Trade openness	495	0.474	0.413	0.017	03.075
Government share	522	0.143	0.054	0.011	0.400
Inflation	544	0.165	0.324	−0.073	2.636
Rule of law	508	0.242	0.937	−1.844	2.000
Commercial bank	495	0.795	0.188	0.126	1.000
Private credit	495	0.363	0.299	0.014	1.803
Liquid liabilities	458	0.782	5.759	0.015	121.7∗
Stock market	233	0.345	0.447	0.000	2.659

Omitting this outlier does not change the results significantly. Note the descriptive statistics are for the updated sample only. Descriptive Statistics for the original dataset are available upon request.

∗ Denotes the outlier Venezuela 1962.

**Table 1:** Comparisons between the two samples.

	Dollar and Kraay (2002)		Updated sample	
	Obs.	Cross sections	Obs.	Cross sections
Basic specification	269	85	414	115
Commercial bank	232	76	367	108
Private credit	221	74	362	109
Liquid liabilities	204	65	332	103
Stock market	53	30	143	69

**Table 2:** Simple growth regressions.

	Income quintile 1	Income quintile 1	Differenced income quintile 1	Differenced income quintile 1	System
	OLS	IV	OLS	IV	GMM
Intercept	−1.762∗∗∗	−2.720∗∗			−1.259∗∗∗
	(0.211)	(1.257)			(0.501)

GDP	1.072∗∗∗	1.187∗∗∗	0.983∗∗∗	0.904∗∗∗	1.012∗∗∗
	(0.024)	(0.151)	(0.079)	(0.119)	(0.060)

P-Ho *β* = 1	0.003	0.215	0.834	0.421	0.836
P-OID				0.177	0.235
T-NOSC					−0.800
Obs.	269	269	269	269	269

Dependent variable = ln Per Capita Income Growth of the Poor. Standard errors in parenthesis. ^∗^ Indicate 10% significance.

∗∗ Indicate 5% significance.

∗∗∗ Indicate 1% significance.

**Table 3:** Simple growth regressions – updated sample.

	Income quintile 1	Income quintile 1	Differenced income quintile 1	Differenced income quintile 1	System
	OLS	IV	OLS	IV	GMM
Intercept	−1.567^∗∗∗^	−4.029^∗∗∗^			−1.494^∗∗∗^
	(0.124)	(1.322)			(0.182)

GDP	1.044^∗∗∗^	1.345^∗∗∗^	0.980^∗∗∗^	1.065^∗∗∗^	1.040^∗∗∗^
	(0.145)	(0.161)	(0.049)	(0.100)	(0.023)

P-Ho *β* = 1	0.003	0.033	0.690	0.517	0.080
P-OID				0.141	0.579
T-NOSC					0.630
Obs.	414	414	414	414	414

*Notes*: As Table 2.

**Table 4:** Growth rates varying by region.

	Dollar and Kraay	Updated sample
Intercept	−4.308∗∗∗	2.437
	(1.421)	(1.737)

GDP	1.355∗∗∗	0.641∗∗∗
	(0.153)	(0.182)

GDP^∗^EAP	−0.413∗∗	0.327∗
	(0.173)	(0.182)

GDP^∗^ECA	−0.290	0.386∗∗
	(0.474)	(0.188)

GDP^∗^LAC	−1.019∗∗∗	0.449∗∗
	(0.368)	(0.193)

GDP^∗^MENA	−0.243	0.399∗∗
	(0.285)	(0.183)

GDP^∗^SA	−0.239	0.390∗∗
	(0.188)	(0.186)

GDP^∗^SSA	−0.230	0.233
	(0.256)	(0.202)

P-Ho *β* = 1	0.020	0.049
[1ex] P-OID	0.133	0.461
T-NOSC	1.571	1.09
Obs.	269	414

Dependent variable = ln Per Capita Income Growth of the Poor. Standard errors in parenthesis. *Notes*: Regional Dummies are included in the regression.

∗ Indicate 10% significance.

∗∗ Indicate 5% significance.

∗∗∗ Indicate 1% significance.

**Table 5:** Additional control variables.

	Openness	G’ment cons	Inflation	Rule of law	Openness	G’ment cons	Inflation	Rule of law
Intercept	−0.858	−1.065^∗^	−0.963	−0.642	−0.969^∗∗∗^	−0.802^∗∗∗^	−0.782^∗∗∗^	−0.445
	(0.705)	(0.580)	(0.594)	(0.602)	(0.344)	(0.280)	(0.282)	(0.365)

GDP	0.993^∗∗∗^	1.019^∗∗∗^	1.002^∗∗∗^	0.950^∗∗∗^	0.998^∗∗∗^	0.990^∗∗∗^	0.979^∗∗∗^	0.931^∗∗∗^
	(0.078)	(0.065)	(0.063)	(0.070)	(0.037)	(0.032)	(0.030)	(0.043)

Openness	−0.039				−0.016			
	(0.150)				(0.058)			

G’ment cons		−0.568				−0.503		
		(0.461)				(0.408)		

Inflation			−0.135				−0.074	
			(0.154)				(0.055)	

Rule of law				0.082				0.071
				(0.062)				(0.046)

P-Ho *β* = 1	0.850	0.772	0.975	0.481	0.961	0.745	0.481	0.111
P-OID	0.870	0.869	0.585	0.486	0.796	0.712	0.757	0.677
T-NOSC	−0.55	−0.40	−0.22	−0.76	1.18	1.09	0.97	−0.02
Obs.	223	237	253	268	359	374	413	362

*Notes*: As Table 4.

**Table 6:** Analysing financial development.

	Commercial bank	Private credit	Liquid liabilities	Stock market	Commercial bank	Private credit	Liquid liabilities	Stock market
Intercept	−0.964	−1.072	−0.799	−0.381	−1.112^∗∗^	−1.124^∗^	−0.985^∗∗^	−2.330^∗∗^
	(0.675)	(1.429)	(0.945)	(1.519)	(0.543)	(0.591)	(0.473)	(1.092)

GDP	0.997^∗∗∗^	1.031^∗∗∗^	0.955^∗∗∗^	0.923^∗∗∗^	0.940^∗∗∗^	1.016^∗∗∗^	0.949^∗∗∗^	1.099^∗∗∗^
	(0.088)	(0.191)	(0.141)	(0.301)	(0.061)	(0.069)	(0.062)	(0.084)

Commercial bank	0.036				0.134			
	(0.212)				(0.280)			

Private credit		0.089				0.214		
		(0.146)				(0.184)		

Liquid liabilities			0.018^∗^				0.023^∗∗^	
			(0.011)				(0.010)	

Stock market				−0.266^∗∗^				−0.156
				(0.103)				(0.095)

P-Ho *β* = 1	0.970	0.871	0.752	0.797	0.322	0.821	0.414	0.235
P-OID	0.832	0.324	0.544	0.416	0.309	0.756	0.650	0.111
T-NOSC	−0.55	−0.46	−0.14	0.10	0.26	0.68	0.78	1.06
Obs.	219	205	189	53	301	292	266	110

*Notes*: As Table 4.

**Table 7:** Analysing finance and regional interactions.

	Commercial bank	Private credit	Liquid liabilities	Stock market
GDP	0.937^∗∗∗^	1.008^∗∗∗^	1.026^∗∗∗^	0.935^∗∗∗^
	(0.056)	(0.066)	(0.106)	(0.080)

Commercial bank	0.538^∗∗^			
	(0.262)			

Private credit		0.354^∗^		
		(0.194)		

Liquid liabilities			0.244^∗∗^	
			(0.096)	

Stock market				−0.011
				(0.074)

Fin^∗^EAP	−0.144	−0.232^∗^	−0.219^∗∗^	−0.242^∗∗^
	(0.124)	(0.134)	(0.092)	(0.112)

Fin^∗^ECA	0.077	0.686^∗∗∗^	0.523^∗∗∗^	−0.076
	(0.120)	(0.237)	(0.201)	(0.322)

Fin^∗^LAC	−0.754^∗∗∗^	−1.540^∗∗∗^	−1.426^∗∗∗^	−0.957^∗∗∗^
	(0.112)	(0.240)	(0.326)	(0.328)

Fin^∗^MENA	−0.142	−0.163^∗∗^	−0.155	0.245
	(0.169)	(0.083)	(0.130)	(0.232)

Fin^∗^SA	0.105	0.929^∗∗^	0.516^∗^	1.248^∗^
	(0.169)	(0.364)	(0.279)	(0.668)

Fin^∗^SSA	−0.474 ^∗∗^	−1.039^∗∗∗^	−0.808^∗∗^	−0.255^∗∗^
	(0.200)	(0.345)	(0.407)	(0.117)

P-Ho *β* = 1	0.929	0.879	0.924	0.026
P-OID	0.564	0.891	0.806	0.426
T-NOSC	0.16	0.66	0.70	0.78
Obs.	301	292	266	110

*Notes*: As Table 4. Intercept and control variables also included in the regression.

## Appendix A

See Tables A and B.

## Footnotes

1 Recent updates state the new poverty line is $1.25 a day as suggested by Ravallion et al. (2008) “Dollar a day revisited.”

2 Financial development is defined in the next section, as are the ways it is measured.

3 www.worldbank.org/research/growth.

4 For the new waves of data I use and agglomerate the UN-WIDER Inequality Database http://www.wider.unu.edu/research/Database to calculate the new income shares.

5 As there is limited data on further measures of the poor such as the headcount ratio it may not be worthwhile examining these variables as observations would be extremely low.

6 Liquid liabilities is measured as M3 as a ratio of GDP and is also known as broad money. It measures the overall size of the banking system. Hence it shows the extent of the formal financial intermediary sector relative to economic activity.

7 For the Dollar and Kraay sample using Stock Market Capitalisation led to a sample of only 53 observations, with Value Traded this figure was far smaller.

8 When the correlations were examined prior to running the regressions, it was found that including both rule of law and schooling would cause multicollinearity problems, and as I was following Dollar and Kraay (2002) more specifically than Beck et al. (2007), I chose the former.

9 The reasoning for using this methodology is to prevent the sample to be over-dominated by those countries whose income distribution data is more frequent and abundant.

10 Full descriptive statistics of the variables may be found in the appendix, Table B.

11 See Beck et al. (2007) for detailed information on this technique.

12 Note that here the *α*and the *μ*_*i*_term get differenced away.

13 Beck et al. (2007) treat financial development as endogenous and instrument it using absolute latitude of a nation’s capital indexed between 0 and 1, and by legal origin.

14 Dollar and Kraay (2002) fail to mention about the rule of law but one would expect that this variable may be endogenous.

15 When instrumenting for these variables in the final regressions, the instrument count becomes almost double to the number of cross sections and GMM becomes inconsistent, as the number of instruments becomes too large.

16 It is also stated that the number of instruments used should be far less than the number of cross sectional units.

17 The control group is Western Europe, USA and Canada.

18 This particular results is very sceptical due to the small sample size.

19 It must be noted that these coefficients represent the values for the control group.

20 Commercial Bank Assets, Private Credit and Liquid Liabilities are the bank based measures of financial development used in the paper.

21 This may be attributable to the Bel-India problem.

22 This debate has also been opened in light of the financial crisis where loan officers of the “big-banks” were predicted to not have screened their clients adequately.
